# Social Media Campaigns: A Game Changer for the Prevention of Breast Cancer in Romania

**DOI:** 10.3390/healthcare12080865

**Published:** 2024-04-22

**Authors:** Raluca Dania Todor, Gabriel Brătucu, Adina Nicoleta Candrea, Christian Gabriel Strempel, Costin Vlad Anastasiu

**Affiliations:** 1 Department of Marketing, Tourism Services and International Affairs, Faculty of Economic Sciences and Business Administration, Transilvania University of Braşov, Colina Universității Street, no. 1, Building A, 500068 Braşov, Romania; raluca.todor@unitbv.ro (R.D.T.); gabriel.bratucu@unitbv.ro (G.B.); christian.strempel@student.unitbv.ro (C.G.S.); 2 Department of Medical and Surgical Specialties, Faculty of Medicine, Transilvania University of Braşov, B-dul Eroilor 29, 500036 Brașov, Romania; costin.anastasiu@unitbv.ro

**Keywords:** Romania, breast cancer, awareness, prevention, social media

## Abstract

Social media can be used to raise awareness about health issues, especially concerning the importance of periodical screening. The present study aims to identify the role of social media awareness campaigns in the early detection of breast cancer, with a focus on Romania, a country with a high female mortality due to this disease. The research is performed using a survey, based on an online questionnaire, which was self-administered by the respondents included in two samples selected from a Facebook community of Romanian women. Sample A was composed of 1945 women who were not exposed to periodic campaigns regarding the importance of screening for the prevention and early detection of this type of disease, and Sample B was composed of 289 women who were exposed to such annual campaigns within the last 5 years. The results outline several differences among respondents from the two samples regarding their awareness of prevention necessity, as well as the frequency and chosen methods for breast cancer screening. The findings hold important implications for public authorities, which must intensify their efforts in raising women’s awareness regarding the importance of screening for the early detection and prevention of breast cancer.

## 1. Introduction

Previous research estimated that approximately one out of every twelve women will develop breast cancer in their lifetime [1]. The highest occurrence of this disease is observed among women aged 45–65 years, coinciding with hormonal fluctuations during the pre- and postmenopausal periods [1]. The various cancers include invasive ductal carcinoma, papilloma, medullary carcinoma, phyllode tumors, ductal carcinoma in situ, lobular carcinoma in situ, and Paget disease [2]. This type of cancer continues to be a challenge for health systems around the world due to the physical, financial, and emotional effects it causes; the treatment of this type of cancer incurs much higher costs than its prevention [3].

Despite the fact that for breast cancer, the 5 years average survival rate is 89.2% overall, the diagnosing stage has a great influence, varying from 98% to 24% survival rate, depending on if it is detected in stage I or stage IV [4]. However, in high-income countries, breast cancer mortality follows a downward trend due to scientific research and improvement treatments, corroborating the increased implementation of early-diagnosis and screening programs. At the European level, the breast cancer screening program is based on performing every 2 years a mammography for asymptomatic women aged between 50 and 69 years old. This screening type is the most widely available test in order to diagnose breast cancer, notwithstanding the asymptomatic or localized stages of the disease [5].

Although prevention via screening is particularly important, it should be mentioned that there can be several types of barriers in the way that can affect positive results over time, like decreasing the mortality rate. A study [4] conducted in 2023 revealed that such barriers could be inclusion in certain ethnic groups (47%), low-income economies (35.3%), level of education (29.4%), no familial cancer history and single marital status (29.4%), medical mistrust and a gap in medical information (23.5%), absence of private health coverage (17.6%), and not carrying annual health checks (17.6%).

### 1.1. General Considerations Regarding Breast Cancer

Breast cancer is the result of the uncontrolled growth of epithelial cells originating in the ducts or lobules of the breast [6]. This condition has become the most common type of invasive cancer and represents the second leading cause of cancer-related death in women.

In 2020, a total of 19,292,789 cancer cases were recorded globally, with breast cancer accounting for 11.7% of the total [7], ranking it at the top of the global list in terms of diagnoses for this type of neoplasm. In Europe, in 2022, breast cancer was the most frequently diagnosed cancer in women, representing 29.4% of the total cancer cases. Thus, one in twelve women is predisposed to developing breast cancer [8]. The highest incidences based on the age-standardized incidence rate (ASIR) calculated in 2020 per 100,000 were in Belgium (113.2), Netherlands (100.9), Luxembourg (99.8), and France (99.1). Also, large incidence rates can be found in Central European countries [9].

One of the main causes for the onset of breast cancer is mutations or defects in the BRCA genes, significantly increasing the risk of breast, ovarian, prostate, or other types of cancer [10]. These genes play a crucial role in DNA damage repair, regulating the cell cycle, and maintaining genomic stability. The absence or impairment of these genes leads to DNA mutations, in some cases progressing to cancer.

When comparing sporadic tumors with those related to the BRCA gene, the latter exhibit an elevated risk of medullary histopathology, also having a higher histologic grade. BRCA1-related tumors fall into the category of triple-negative breast cancer, overlapping with basal-like breast cancer.

However, genetic mutations in the BRCA gene do not represent the only cause of breast cancer. Previous studies [11] have revealed that both genetic factors and lifetime exposure to estrogen are involved in the development of breast cancer. The formation of breast tumors is influenced by both the effects of the estrogen receptor alpha and non-receptor mediated effects of estradiol (E2). By stimulating the growth of the cells, estrogen contributes to the development of cancer via the estrogen receptors.

### 1.2. The Importance of Social Media in Breast Cancer Prevention

Actual studies show that one in three people use social networks worldwide [12]. The trend among social media users suggests that specialists believe this upward trend will continue. Social media is composed of various platforms that facilitate the dissemination of compelling content and the creation of dialogue, thereby communicating to a broader audience [13]. Social media offers a favorable environment for interacting and networking about relevant topics for each user and for all levels of society, serving as a space created by people for people in the digital era. Social media, a term encompassing various online platforms and tools, enables the sharing and generation of content [14]. Within this realm, social networks form a subset, emphasizing the establishment and upkeep of connections among individuals and groups [15].

The use of social networks made it a lot easier for users to access content that is relevant to them. Whether the curation is made by the user or by the specific algorithms [16], social platforms ensure that users receive the information that fits them.

Social networks provide tools for much faster dissemination of information. A significant innovation offered by social networks compared to traditional methods of information dissemination is the ability to create discussions and groups, fostering a bidirectional relationship [17]. In Romania, there are 13.5 million active users of online social media networks, which equates to 67.3% of the total population. According to the same study, a total of 70.75% of this population (9.55 million inhabitants) use Facebook [18]. This bidirectional communication aids in the creation of communities centered around various topics, such as breast cancer, for example. By establishing communities with an interest in this subject, dissemination of information becomes much more accessible, easily reaching the end user. Moreover, given the speed at which information spreads through these networks, it can also be utilized for preventive campaigns.

Social media has become a significant and popular medium for disseminating health-related information, and it offers creative means of raising public awareness and comprehension of cancer prevention messages [19]. The potential of social media to engage audiences for better communication and increased capacity to promote programs, products, and services should be valued when using it for health promotion [20]. Social media networks have the power to inform people about health issues in novel ways. The explanations for this are their ability to spread content virally, support and encourage social interaction, alter norms, and perhaps impact behavior change [21]. The engagement of the platforms attains the goal of each campaign [22], namely information dissemination of important issues.

Healthcare prevention via social media might be an efficient tool for reaching a wide audience and sharing valuable information. Previous studies [23] have revealed that the chosen channel is very important for developing an efficient campaign for the prevention of various diseases, such as breast cancer.

Because people can now search and share information on multiple sources, messages about breast cancer are no longer only transmitted via traditional media channels [24]. One way to raise women’s awareness regarding breast cancer is the use of social media that enables followers to spread health-related information easily and consistently. Social media such as Facebook, Twitter, YouTube, and Instagram provide users with unique mechanisms to transmit and receive information and feedback in different forms of rating symbols, comments, and shares [25]. Among these networks, Facebook is the most popular.

Breast cancer is present on social media [26], just like in everyday life. Given that it is such a widespread disease, it would have been surprising for it to be absent here. For breast cancer prevention, it is crucial to combat misinformation. Creating prevention health campaigns can be the solution [27]. Additionally, targeting specific age groups is also a very important aspect of creating the campaign because, via the early detection [28] and discovery of breast cancer, it can be fought more efficiently.

Educating young women about their breast cancer risks, symptoms, and self-detection has the potential to increase risk reduction behaviors across the age trajectory, increase earlier detection, and promote overall health and well-being [29].

Creating a community is a very important part of the entire activity because this is the core of the prevention campaign [30]. Growing communities have great potential for delivering health support [31]. Fostering a sense of connection and sharing the same purpose, these communities can serve as valuable platforms for disseminating information, offering assistance, and promoting well-being. Sharing experiences and knowledge has a crucial role in strengthening community bonds.

A shortcut to creating prevention campaigns can be the use of social media influencers. Even though influencers are not specialists in healthcare policies or prevention campaigns, they demonstrate greater engagement with users from specific countries. More than that, the use of a female influencer for the development of a prevention campaign might increase the effectiveness of the campaign because of the choice of image for the cause, which aligns with everyday topics [32].

### 1.3. Study Context—Breast Cancer in Romania

Breast cancer is the most common type of cancer among women globally, and Romania is no exception to these statistics [33]. In Romania, within the total estimated population of 19 million inhabitants, women account for approximately 51.6%, equating to an estimated 9.8 million female inhabitants. Regarding life expectancy, Romania is situated in 91st place of the worldwide ranking, below the European Union life expectancy of 80.7 years for the total population. Romania has a general life expectation of 75.6 years, which is below the European Union average by 5.4 years [34]. Unfortunately, according to World Life Expectancy, Romania ranks 20th in total deaths due to cancer, with 141.06 deaths per 100,000 inhabitants [35]. Breast cancer ranks Romania 68th worldwide in terms of death rate, with 18.79 deaths per 100,000 inhabitants in 2023, also being the seventh cause of mortality in Romania. One of the most particularly important factors that led to this situation is the lack of national programs to prevent this type of cancer. Moreover, studies show that in Romania [36], education regarding health and prevention is particularly poor, and the main causes of mortality shown in Table 1 confirm this fact since many of them are preventable kinds of diseases.

A previous study carried out on the female population from Romania [37] demonstrated that education plays an essential role when it comes to the behavior of women toward screening investigation. In addition, the study highlights that the overall female population in Romania has the lowest participation in screening investigations in the European Union. Unfortunately, the Romanian Ministry of Health has not yet implemented any free breast cancer screening campaigns. Therefore, raising women’s awareness regarding this cancer has become an imperative measure in the attempt to reduce its incidence in Romania.

Despite the high mortality rate due to this type of cancer, relatively few studies have been carried out on the impact of breast cancer on the female population in Romania, on topics such as breast cancer mortality gaps in Romanian women compared to the EU countries [36], the importance of information and education campaigns, risk factors and prophylaxis [33], the role of cause-related marketing in the case of breast cancer in Romania [38], breast cancer screening and prophylactic mastectomy for high-risk women in Romania [39], etc. However, no study was conducted previously that measures the impact of social media campaigns on the level of awareness regarding the importance of screening and the advantages of early diagnosis of breast cancer. Attentive to the importance of educating women about the risks of breast cancer and the role social media can play, this paper aims to fill this gap in the existing literature focused on this particular country setting. The main objective is to determine the extent to which education via social media campaigns regarding the importance of breast cancer screening prevention could determine more responsible behavior of women by periodically undergoing routine checks for the early detection of this type of disease. To achieve this main objective, the research has a series of specific objectives, which are listed as follows:

O1: Finding the extent to which women know about the importance of screening for breast cancer prevention.

O2: Identification of the types of used control methods.

O3: The frequency with which women visit a doctor for a routine check-up regarding breast cancer.

O4: Identifying the main reasons why women do not make these periodic check-ups.

O5: Finding the differences between women who were periodically exposed to informational campaigns in social media for 5 years and those who were not regarding their awareness and behavior for breast cancer prevention.

## 2. Materials and Methods

As previously mentioned in this paper, the Romanian Ministry of Health has not yet implemented any national prevention and screening programs for breast cancer, a fact that can explain, to a certain extent, the current situation regarding the incidence and mortality rate caused by this type of cancer. On the other hand, in Romania, the only existing information and education campaigns were organized by private healthcare institutions, non-governmental agencies, or by volunteers who targeted large groups on social media.

Such periodic campaigns were carried out within the largest community of mothers in Romania: the Facebook group “La primul bebe”. Founded in 2012, this online female community represents the largest community of educated women in Romania, with approximately 250,000 members. Facebook-awarded and supported back in 2018, this community, together with 99 other communities from all over the world, has a significant impact on society. These 100 communities were selected out of 6000 communities that entered the competition to be part of the Facebook Community Leadership program [40]. One of the most important achievements of this community is that it implemented educational and awareness-raising campaigns regarding the importance of screening for the prevention of breast cancer [41].

The present study uses a quantitative research approach based on a survey applied to Romanian women who are members of a social media group. Primary data were collected from respondents via a questionnaire (see Appendix A) that was distributed online via the online Facebook community “La primul bebe”. The used data collection method is purposive sampling, so the results cannot be generalized for the entire female population in Romania. The data resulting from the administration of the questionnaire were then processed separately for two samples, with the purpose of outlining the differences in the awareness of the need for breast cancer screening. A filter question was applied in the first part of the questionnaire regarding women’s exposure to periodic breast cancer awareness campaigns, with the purpose of framing each respondent in one of the two samples. Sample A was composed of 1945 women who were not exposed to periodic campaigns regarding the importance of screening for the prevention and early detection of this type of disease, and Sample B, composed of 289 women who were exposed to such annual campaigns in the last 5 years; all were members of the community “La Primul Bebe”. A survey was carried out between May and September 2023 using a Google Forms questionnaire, and the resulting data were analyzed using Microsoft Excel. The samples were made by random sampling. The participants in both samples were women from the 14 largest cities of Romania: Bucharest, Ploiesti, Brasov, Cluj, Constanta, Iasi, Timisoara, Galati, Braila, Craiova, Sibiu, Bacau, Pitesti, and Suceava. The structure of the two samples is presented in Table 2 by age, level of income, and level of education.

The resulting data were analyzed in a comparative manner for the two samples (A and B), with the purpose of outlining a potential connection between respondents’ behavior for the prevention of breast cancer (especially regarding the frequency and type of screening methods) and their exposure to periodic campaigns regarding the importance of screening for the prevention and early detection of this type of disease.

## 3. Results and Discussion

A first step in the research process was to ask the surveyed women to what extent they are aware of the importance of screening for breast cancer. The results of the research show that there is an important difference, from this point of view, between the two surveyed samples, which is explained as follows:

Sample A: A total of 59.79% (1163) women from Sample A (who were not periodically exposed to information campaigns in social media) stated that they were aware of the importance of screening, while 40.21 of them (782 women) declared that they had no knowledge in this regard.

Sample B: Meanwhile a percentage of 87.54% of respondents from this sample, meaning 253 women (who were periodically exposed to informational campaigns in social media for 5 years), reported they were aware of the importance of breast cancer screening, while only 12.46% were not aware of the importance of breast cancer screening.

**Figure 1:**
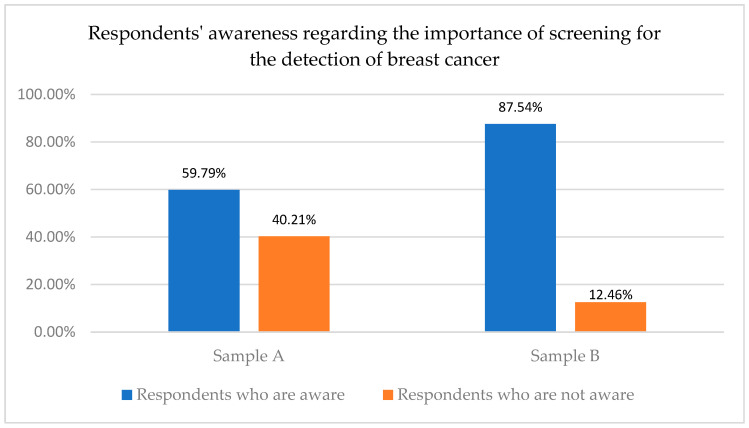
Respondents’ awareness regarding the importance of screening for the detection of breast cancer.

This implies that the majority of the respondents from each sample were aware of breast cancer disease, which is in line with previous studies [42]. Considering this outcome, the first objective of the study was attained (O1). However, based on the comparative analysis of respondents’ awareness regarding the importance of screening for the detection of breast cancer, we can observe that there are significant differences between women who were periodically exposed to informational campaigns on social media and those who were not (Figure 1). Therefore, we may consider the potential role of exposure to educational campaigns performed via social media in increasing women’s awareness regarding the importance of screening for the early detection of breast cancer, an issue that helps meet the fifth objective of this research (O5).

**Figure 2:**
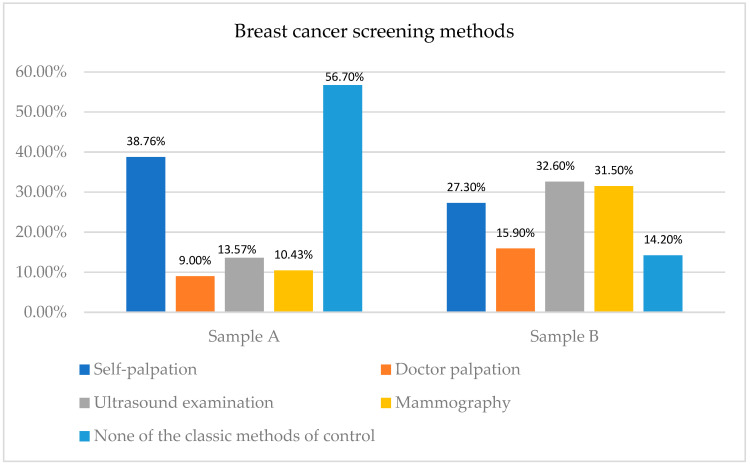
Types of methods used for periodical breast cancer screening.

The second objective of this study (O2) was to identify the types of control methods used for breast cancer screening by the respondents. The types of methods used by the respondents from the two analyzed samples for periodical breast cancer screening are depicted in Figure 2, responding to the second (O2) objective of this paper. Self-palpation is a popular screening method in both samples (more frequent in the case of women in Sample A), as well as ultrasound examination.

In spite of the acknowledged awareness of breast cancer disease identified among the majority of the respondents from each sample, many respondents from each sample do not attend or perform clinical breast cancer examinations, an outcome which is in line with previous research (e.g., Wogu et al., 2019 [42]).

Moreover, when respondents were asked which method they choose to carry out periodic medical checks of their breasts, the research results indicated certain differences in the two analyzed samples:

Sample A: 38.76% (754 women) use self-palpation as a control method, 9% (175 women) choose palpation performed by the doctor as the method, while 13.57% (264) respondents) opt for an ultrasound examination, and only 10.43% (203 women) choose mammography as the screening method.

Sample B: 79 women, representing 27.3%, choose self-palpation as the control method, while 46 of them (15.9%) choose palpation by the doctor as the method. An amount of 96 women (32.6%) consider ultrasound to be the ideal control method, and 91 women (31.5%) undertake a mammogram periodically.

A significant difference among samples can be observed in the case of women interviewed in this sample who do not use any methods of screening for breast cancer: 56.7% in Sample A (who were not periodically exposed to information campaigns in social media) and 14.2% in Sample B (women who were periodically exposed to information campaigns in social media).

**Figure 3:**
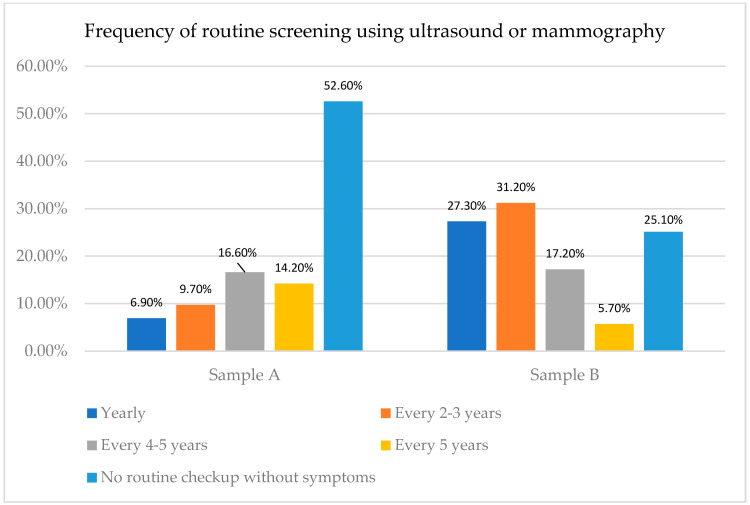
Frequency of periodical screening using ultrasound or mammography.

Another objective of this study aimed to establish the frequency with which women visit the doctor for routine check-ups to perform an ultrasound or mammography (O3). The research results show the following (Figure 3):

Sample A: only 6.9% of the respondents of this sample present themselves at the ideal interval, namely one time per year; 9.7% of them perform a periodic checkup every 2–3 years; while 16.6% present only once every 4–5 years; and 14.2% even less often, once every 5 years.

Sample B: a higher percentage, specifically, 27.3%, of the respondents, opt to have annual routine checkups, choosing either ultrasound or mammography as the screening method. For Sample B, 31.2% choose to undertake a routine check-up once every 2–3 years, while 17.2% only see a doctor once every 4–5 years for this kind of screening; and 5.7% of respondents perform a routine check-up less often than once every 5 years.

These outcomes clearly show that women who have been exposed to periodic informational campaigns in social media visit the doctor more frequently for these routine checks, thus responding to the fifth objective of the present study (O5). Furthermore, this confirms previous studies, which highlighted the role of social media tools in cancer prevention [43].

**Figure 4:**
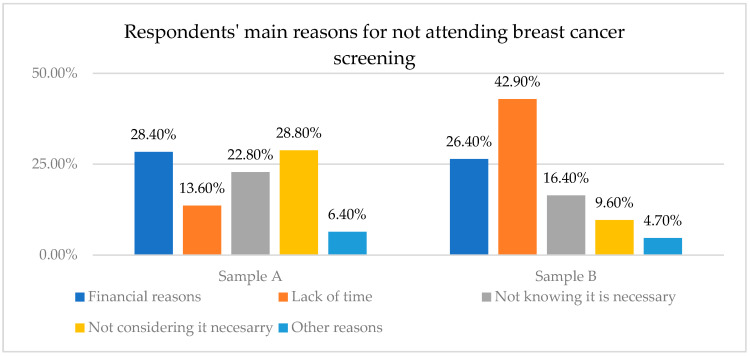
Respondents’ main reasons for not attending breast cancer screening.

The fourth objective of this study aimed to identify the main reasons why women do not undertake periodic breast cancer screening (O4). The following results have been registered for each sample (Figure 4):

Sample A: The research shows that 56.7% of women interviewed in this sample do not use any of the classic methods of control for breast cancer screening. Moreover, the findings outline the main motivations for this choice: financial reasons (28.4%), lack of time (13.6%), not knowing it was necessary (22.8%), not considering it was necessary (28.8%), and other reasons (6.4%).

Sample B: 25.1% of the respondents of this sample say that they do not usually have routine checkups for the detection of breast cancer if they do not have any symptoms. Respondents from Sample B (who were periodically exposed to information campaigns in social media) indicated the following reasons for not having periodic checks: lack of time (42.9%), lack of financial resources (26.4%), not knowing it was necessary (16.4%), not considering that it is necessary (9.6%), and other reasons (4.7%).

An important outcome is that more than half of the respondents from Sample A, composed of women who have not been exposed to periodic informational campaigns in social media, stated that they do not usually undertake a routine checkup if they do not have any symptoms. In contrast, this percentage is much lower in the case of respondents who were periodically exposed to information campaigns on social media. This significant difference between the two analyzed samples (contributing to meeting O5) may account for the exposure of women from Sample B to informational campaigns in social media regarding breast cancer prevention. This outcome helps attain the fifth objective of the study (O5) and is in line with the results of a previous study, which suggested that that social media intervention may improve cancer screening and early diagnosis [19].

## 4. Conclusions

The main purpose of this study was to determine if exposure to education and responsibility campaigns via social media can lead to a change in behavior regarding breast cancer prevention. Several outcomes were identified based on the results that emerged from the survey conducted among Romanian women who were or were not exposed to breast cancer awareness campaigns in social media within the last 5 years.

The first finding of this study revealed differences among the two analyzed samples concerning respondents’ awareness of the importance of breast screening. Therefore, we found that women who were periodically exposed to informational campaigns in social media within the last 5 years have a high level of awareness regarding the necessity of screening in the early detection of breast cancer. Furthermore, the outcomes indicate that women who have been exposed to periodic informational campaigns in social media visit the doctor more frequently for screening routines aimed at detecting breast cancer. This may indicate the role of social media campaigns in raising women’s awareness regarding the role of such screening initiatives.

Furthermore, the outcomes show certain differences between the two samples concerning the choice of method for breast cancer screening. Most respondents (almost half of each sample) reported self-palpation as a control method. However, this method has the disadvantage of not being performed correctly, and in addition, it is possible that it cannot detect the disease in its early stages. Nonetheless, we found that the use of ultrasound and mammography as breast cancer screening methods is much more frequent in the case of women who were periodically exposed to informational campaigns on social media. A significant difference appears in what concerns the number of women who do not opt for any method, which is 14.2%, significantly lower than in the case of women who were not exposed to preventive campaigns for breast cancer, respectively 56.7%. This indicates that women who are exposed to awareness campaigns have a higher chance of undertaking breast cancer screening. Moreover, the study results indicate that more than half of the analyzed respondents, who were not exposed to social media awareness campaigns (52.6%), stated that they do not usually undertake a routine checkup if they do not have any symptoms, and a very small amount (6.9%) undertakes annual screening procedures for the detection of breast cancer. In the case of female respondents who were exposed to social media awareness campaigns, this percentage is higher (27.3%), which may indicate the role of these initiatives in women’s choice for annual screening.

Moreover, this study reveals the main reasons why the interviewed women do not opt for any screening method. Financial reasons are one of the main motivations in both analyzed samples, in addition to the lack of time and the lack of knowledge regarding the importance of this medical procedure in the early detection of breast cancer. An alarming percentage (28.8%) of women who were not exposed to informational campaigns in social media are not aware of the necessity of breast cancer preventive screening. Nonetheless, almost half of the respondents (43%) who were exposed to informational campaigns on social media mentioned the lack of time for not undertaking breast cancer screening. These findings hold important managerial implications. Although a first step toward raising awareness of breast cancer screening has been undertaken by the NGO “La primul bebe” via its social media campaign, it is far from enough. Romanian medical and governmental bodies must periodically implement such awareness-raising campaigns, targeted at informing the female population in the country regarding the importance and necessity of screening in the early detection of breast cancer, emphasizing the most efficient screening methods. Nonetheless, these authorities, in cooperation with NGOs, need to find funds that can be allocated for breast cancer screening in the case of women with low revenues or special social cases.

Although this study provides an overview of the role of social media campaigns in breast cancer screening prevention, it has several limitations. First, it is only focused on one country (Romania). Cross-country comparisons could be performed in future studies, which may analyze the differences among women from developed or developing countries or even from the EU or other regions of the world. Second, social desirability might have influenced women’s responses regarding the frequency and type of screening methods, as well as the motives for not undertaking breast cancer detection procedures. Third, the data selection is not based on a random sampling method; therefore, the results cannot be generalized for the entire female population in Romania. Future studies may consider carrying out, in the first phase, some qualitative research of the “group interview” type (focus group), which will generate the information needed to formulate the objectives and general hypotheses of a new quantitative marketing research. Furthermore, it would be necessary to generate a representative sample as it will be based on a random/probability sampling method using a face-to-face data collection method with the help of interviewers.

## Figures and Tables

**Table 1:** Main causes of death in Romania.

	Cause of Death	Number of Deaths/100.00	World Rank
1	Coronary Heart Disease	153.78	46
2	Stroke	104.36	65
3	Lung Cancers	28.30	18
4	Liver disease	27.34	66
5	Influenza and Pneumonia	26.33	96
6	Hypertension	22.29	71
7	Breast cancer	18.79	68
8	Colon–Rectum Cancers	17.21	11
9	Lung Disease	16.92	127
10	Prostate Cancer	13.75	114

Source: World Life Expectancy https://www.worldlifeexpectancy.com/country-health-profile/romania (accessed on 30 November 2023).

**Table 2:** Samples structured by age, education, and income level.

Characteristic		Sample A	Sample B
		Percent	Percent
Age	20–30	574	82
		29.50%	28.40%
	30–40	1310	126
		67.30%	43.60%
	40–50	55	61
		2.80%	21.20%
	50–60	6	20
		0.40%	6.80%
Total		1945	289
		100%	100%
Level of education	High School	356	14
		18.30%	4.80%
	Bachelor’s Degree	916	147
		47.10%	50.90%
	Master’s degree	673	128
		34.60%	44.30%
Total		1945	289
		100%	100%
Level of income	2000–3500	191	-
		19.40%	0%
	3500–5000	325	57
		27%	19.40%
	5000–6500	533	78
		23.40%	27%
	6500–8000	263	84
		10.10%	29.40%
	8000–9500	232	38
		9%	13.10%
	Over 9500	401	32
		11.10%	11.10%
Total		1945	289
		100%	100%

## Data Availability

The data presented in this study are available upon request from the corresponding author. The data are not publicly available due to privacy reasons.

## Appendix A. Questionnaire

1. Are you aware of the importance of breast cancer screening? □ Yes □ No 2. If you are aware about the importance of screening for breast cancer, how do you usually perform a check-up? □ Self-palpation. □ Palpation by the doctor. □ Ultrasound. □ Mammography. □ None of the above. 3. How often do you see a doctor for a routine ultrasound or mammography? □ Once a year □ Once every 2–3 years □ Once every 4–5 years □ Less often than once every 5 years □ Never if I don’t have any symptoms. 4. What are the main reasons why you choose to undergo periodic screening for breast cancer? □ Existing breast cancer cases in my family or in the entourage. □ This was indicated to me by the doctor. □ I consider it absolutely necessary because I am aware of its benefits regarding the early detection of breast cancer. □ Fear of illness. □ Other …… 5.What are the main reasons you choose NOT to undergo periodic screening for breast cancer? □ I don’t think it’s necessary if I don’t have any symptoms. □ I do not have the financial resources necessary to carry out such a periodic check-up. □ I did not know that it is necessary to carry out these periodic check-ups. □ I don’t trust the results of this check-up. □ I don’t have time. □ Other…. 6. What is your age? □ 20–30 □ 30–40 □ 40–50 □ 50–60 7. What is the last school you graduated from? □ High school □ University studies □ Postgraduate studies 8. What is your income level (in RON)? □ 2000–3500 □ 3500–5000 □ 5000–6500 □ 6500–8000 □ 8000–9500 □ Over 9500
